# The ascendance of microphysiological systems to solve the drug testing dilemma

**DOI:** 10.4155/fsoa-2017-0002

**Published:** 2017-03-31

**Authors:** Eva-Maria Dehne, Tobias Hasenberg, Uwe Marx

**Affiliations:** 1 TissUse GmbH, Oudenarder Straße 16, 13347 Berlin, Germany

**Keywords:** body-on-a-chip, drug testing, human-on-a-chip, microphysiological system, multi-organ-chip

## Abstract

The development of drugs is a process obstructed with manifold security and efficacy concerns. Although animal models are still widely used to meet the diligence required, they are regarded as outdated tools with limited predictability. Novel microphysiological systems intend to create systemic models of human biology. Their ability to host 3D organoid constructs in a controlled microenvironment with mechanical and electrophysiological stimuli enables them to create and maintain homeostasis. These platforms are, thus, envisioned to be superior tools for testing and developing substances such as drugs, cosmetics and chemicals. We will present reasons why microphysiological systems are required for the emerging demands, highlight current technological and regulatory obstacles, and depict possible solutions from state-of-the-art platforms from major contributors.

## Lay abstract

Microphysiological systems are devices constructed for the cocultivation of miniaturized human organ equivalents. They are commonly placed into a continuous stream of nutrient solution. The microphysiological tools aim to reshape current development, toxicity testing and efficacy assessment of therapeutic agents, food additives, chemicals and environmental pollutants. We are on the verge of initiating a paradigm shift away from established, but often misleading animal and single tissue culture techniques toward the generation of predictive data for a compound's safety and efficacy prior to its exposure to humans.

## The drug testing dilemma

Drug developers are facing an ever-increasing problem – their costs for releasing a new product are skyrocketing. The authorization process including clinical and preclinical trials is not only complicated and highly restrained, but takes an exceptionally long time. It is estimated that the development, testing and authorization of a single drug candidate currently takes up to 13.5 years [1] and costs an average of US$ 2.5 billion [2]. The cost explosion over the past decade has naturally several reasons. One major cost driver is the fact that of 100 substances entering clinical testing in humans, only ten will be allowed onto the market [2,3]. The high attrition rate is cross-financed by those few substances. Approximately half of the candidates excluded are retracted due to safety concerns [4,5]. Apart from deprecating an investment, there is an eminent risk for any proband in the clinical trials, as was yet again obvious regarding a fatality in a Phase I clinical trial in early 2016 [6,7]. The regulatory safety requirements are indubitably a main driver for the increase in costs. However, nobody is inclined to sacrifice risk assessment for commercial optimization.

**Figure 1.**
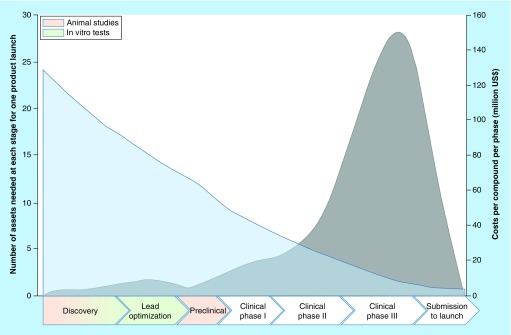
**The drug testing dilemma.** The graph illustrates schematically the route from substance development through preclinical and clinical evaluation of toxicity and efficacy until release. The process is estimated to take an average of 13.5 years. Depending on the source, only 4–10% of the initial drug candidates will become market-ready and will cross-finance the withdrawn substances. Retraction at a late stage becomes especially costly, which is why the developing companies try to improve the predictability in the preclinical phases and resolve inapt drugs (numbers are extracted from Paul *et al*. [1]).

Consequently, the substance testing should be optimized in such a way that candidate drugs are retracted from the risk assessment as early as possible [1]. Therefore, the drug discovery and the preclinical risk assessment are thought to be the prominent levers for reducing costs (Figure 1).

The difficulties for drug developers and toxicologists are now manifold. First, their test systems are obviously not optimal to predict safety and efficacy in humans properly. Simultaneously and second, safety requirements will rather intensify than halt. Third, the amount of substances awaiting testing will increase [8]. Most importantly, their complexity will be boosted as more and more biopharmaceuticals are in development [9,10], along with mixtures that have effects unpredictable from the respective single substance knowledge [11].

Substances are tested currently, first and foremost, either in animal models – mostly rodents – or with human cell cultivation techniques. Toxicologists might refer to them as proxy and reductionist models, respectively [12]. The former permits access to an entire organism with all its immunity, microenvironments, organ–organ interactions and behavioral responses to any given substance. Some might regard them as the gold standard, but they have more than once proven to be inaccurate due to their phylogenetic distance and methodological flaws [13–17]. In fact, widely used drugs, such as aspirin or paracetamol, would not be approved with current animal models [18]. Their predictability is questionable as they are obviously not human. In the words of Marcel Leist and Thomas Hartung: “Humans are no 70-kg mice” [16]. Apart from the candidate drugs that were incorrectly allowed for human testing, there may be substances equally incorrectly repealed waiting to be retrieved like a forgotten treasure.

*In vitro* cultivation – the reductionist model – is usually based on human cells. It is a field of constant innovations and new technologies, especially in the area of tissue engineering. The declared ambition is the emulation of the functionality of any organ. These tissue models are often dedicated for regenerative therapies. They are equally desired in substance testing. Nevertheless, focusing on a specific tissue will not overcome the dilemma described, no matter how close-to-physiological a model becomes. Toxicity often emanates from complex interactions across tissues. The diversity of metabolites is especially regarded as critical. The drug Terfenadine, for example, is only activated in the liver [19]. However, the activated form simultaneously becomes cardiotoxic. Regarding the fatality case mentioned earlier, metabolites of the drug candidate remain a probable cause for the dire outcome of the clinical trial [7].

Moreover, as the cell culture is often a reduced, out-of-context model, it is hard to relate concentrations of substances back to the human case. The exposition *in vivo* is often either unknown or hard to predict [12]. Similarly, the influence of absorption, distribution, metabolism and excretion (ADME) needs to be regarded. The extrapolation of the data gathered to the *in vivo* situation is, therefore, again highly questionable. Hence, conclusions gained from cell culture models usually give rise to yes or no answers rather than dose information.

The dilemma for toxicologist is now the following: while regulations, quantity and complexity of the substances increase continuously, current test systems are partly outdated, partly weak in predictability. The estimation of safe starting and maximum doses for clinical phases in humans are founded on mere leap-of-faith decisions than on well transferable data.

In fact, the issues concerning substance testing are encountered not only by the pharmaceutical industry. In 2013, the European Union banned all animal-based experimentation for newly approved cosmetic products. Additionally, new cosmetics that have been developed and tested in animal-based experiments were excluded from the European market [20]. Although it does not prohibit animal tests, the REACH regulation (Registration, Evaluation, Authorization and Restriction of Chemicals) is also thought to have contributed to the development of alternative tests. It demands transparency of toxicological datasets, encourages alternative methods and tries to prohibit the reassessment of toxic queries in animals by demanding approval by the agency [8,21].

The rise of microphysiological systems (MPS), about a decade ago, marks a turning point. They allowed the combination of highly sophisticated human organ models and mimic their systemic interplay. The variety of solutions and developments underscore the importance and potential ascribed to these devices – not only by scientists, but also by the industrial end users. Other economic and scientific fields, such as food or ecology, will profit similarly from human MPS. If these devices convince industry and regulators alike, they will initiate a paradigm shift away from obsolete *in vivo* experiments toward new approaches of substance development and risk assessment.

## Microphysiological systems in the context of cell culture technology

In short, MPS are microfluidic devices designed to support a physiological environment for *in vitro* cultures and, finally, emulate biology. An MPS in its simplest form resembles, for example, the device published by Heemskerk *et al*. [22]. Here, a perfused channel of 300 × 52 μm lined with endothelial cells emulates a blood vessel's intima. The introduction of a stenosis into the channel simulates plaque formation during atherosclerosis. The increased activity of von Willebrand factor in the stenotic region demonstrates the ability of the MPS to act as a disease model. More complex MPS comprise a network of interconnected microchannels in combination with cocultures of several different organ models [23]. A selection of MPS devices will be presented later.

The MPS are the logical successor of lab-on-a-chip devices ascending at the beginning of the last decade. Different to what the term suggests, these tools do not necessarily comprise microelectronics. Their manufacturing, however, makes frequent use of semiconductor techniques enabling the miniaturization of huge and expensive devices. The MPS do not necessarily profit from the same ease-of-use and point-of-care functionalities. The benefits for MPS lie somewhere else.

The MPS devices, for instance, enable the building up of physiological-like microenvironments for respective cell cultures. In terms of 3D cultivation techniques, the MPS can be designed for hosting and improving such cultures. The perfusion of the systems enacts a proper shear stress environment at physiological intracapillary or interstitial rates. It clears secreted products, permits the interaction of distant cells and the creation of microenvironmental biomolecular gradients. The generation of relevant mechanical cues is a major aspect of these systems, distinguishing them from conventional static *in vitro* cell cultures; for example, the stretching of lung epithelial cells [24] or the electrophysiological stimulation in cardiac models [25]. The drive to catch up with the physiological template will introduce disease and regenerative models [26]. Such endeavors raise the need for an immune system in MPS, even if partial. The systems enable the incorporation of extracellular matrix components or specialized materials, such as ceramics, textiles or polymers, to model stiffness, surface patterns or the microarchitecture.

Furthermore, the miniaturization reduces the amount of cell material and increases the throughput. This is the buzz word for every toxicologist facing an increasing mass of substances in their pipeline. Of course, special care should be taken, as the smallest possible size is not necessarily the most practicable. The MPS engineers are generally interested in subtissue structures – particularly organoids [27].

Organoids are defined either as the smallest functional *in vivo* units of any organ or as the respective functional *in vitro* 3D cell accumulations. The former are identical, functionally self-reliant units realizing the most relevant tasks of the entire organ. They typically comprise a large number of cells, different cell types and a defined microstructure. A prime example of an organoid is the liver lobule. In humans, each lobule contains about 1 million cells, about 20 cell types and a complex vessel architecture. One million lobules constitute the liver. Similar sections can be found in every organ [28]. Tissue engineers intend to reconstruct these organoids in MPS.

The maintenance of the cell cultures within the MPS is further improved by functionalities controlling the temperature, pH and even oxygen [29]. The monitoring capabilities of MPS also allow accurate and continuous observation of the cells. Electrical coupling enables the readout of action potentials in neuronal or cardiac cultures. Transparent bottom plates do not only permit visual insights, but also the optical observation of, for example, oxygen partial pressure [30]. Integrating various sensors and actuators enhances the degree of control substantially.

## Challenges & chances in MPS development

As described above, the overall goal of all MPS is the emulation of species-specific biology. However, the scales, basic working principles and biological complexities are varied. These are designed to fit different purposes, matching the technical solutions available for the design of the tissue culture compartments and the microfluidics.

The MPS can be generally classified into two factions regarding the generation of fluid flow: passive gravity-driven flow and active pumping. The former was developed regarding standardization, automation and high-throughput testing without the need for extensive external accessories and vast medium reservoirs. These devices are often in the form of a microtiter cell culture plate and its cavities are usually in the same locations as in 96- or 384-plates. Depending on the system, several cavities are microfluidically interconnected and the flow is hydrostatically driven (pumpless perfusion).

Platforms comprising active pumping, on the other hand, generally follow a proprietary design concept. Their less standardized format allows more variability in the dimensions of the organoid compartments, complexity of the microfluidics and integration of sensors. Many devices depend on external roller or syringe pumps, others contain on-chip micropumps [24,31–33]. The former benefit from ease of production and operation, but apart from that, include substantial drawbacks. First, the fluid-to-tissue ratios are beyond the normal physiological range due to the artificially high media volumes required for external pumps. Organ–organ crosstalk is, thus, compromised because the soluble factors are strongly diluted. The use of external tubes, adapters and, hence, many different materials also aggravate issues concerning unspecific substance adsorption. On-chip pumps, on the other hand, are difficult to characterize, especially often due to their pulsatile fluid flow generation.

An extensive review of a recent workshop with members of academia, industry and regulators comprehensively summarizes the concepts and examples for various MPS [34]. In the following, the main challenges and opportunities of both MPS types will be discussed based on commercially available or well-reviewed systems.

### The advantages of continuous perfusion

A long-term culture is often impeded due to loss of function, especially when working with demanding primary cells such as hepatocytes. The nonhomeostatic conditions and sudden media changes in static *in vitro* cell cultures often lead to a dedifferentiation of cells. Therefore, single-organ engineers aimed early on for a continuous supply of nutrients and removal of ‘waste’ products that cannot be metabolized by the respective cells and would accumulate potentially harming the cells. Cell culture formats with passive gravity-driven microfluidic flow emerged. The CellASIC Corporation (now part of Merck KGaA, Darmstadt, Germany) was one of the first companies commercializing microtiter plates for continuously perfused monolayer cell cultures. Their system is based on a disposable 96-well plate containing four microfluidic circuits, all sample solutions, media and cells. A network of 4 × 4 μm channels surrounds the entire culture compartment. It maintains the cultured cells in a specific imaging region with exposure to continuous media perfusion [35]. The technology was used previously in a custom-made fluidic layout to cultivate hepatocytes under high-density for over 1 week [36]. The continuous medium flow did not only keep cells viable, but also enhanced the specific production of albumin by threefold compared with static cultures. The physiologically relevant nutrient supply and flow dynamics provided the necessary cues for maintaining the differentiated phenotype and function of cells. This study further demonstrated the importance of high cell densities.

Cell–cell communication is essential for cells to sense and respond to their environment. Drug toxicity often disrupts intercellular connections, initiating a cascade of cellular events that leads to loss of function and apoptosis. Therefore, recapitulating cell–cell interactions analogous to their *in vivo* counterparts is critical for physiologically relevant organ models. However, the cell density under standard *in vitro* conditions is usually 100- to 1000-fold lower than in tissues. Moreover, the commercially available CellASIC^®^ plates are not intended for high cell density culture.

### Recognizing the relevance of 3D cocultures & physical stimulation

**Figure 2.**
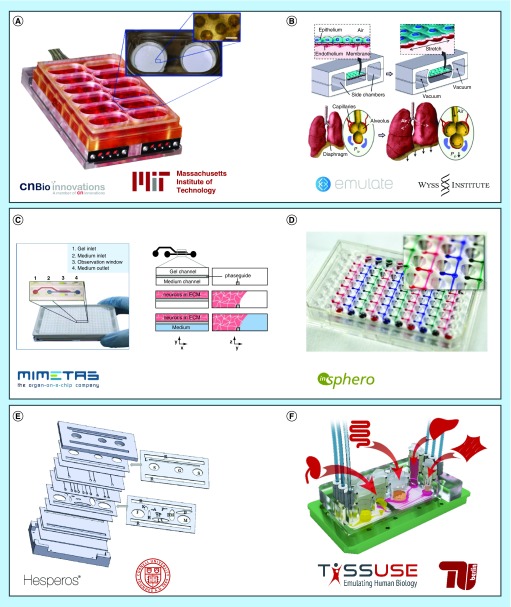
**Selection of current microphysiological systems devices with ongoing marketing or commercialization ambitions.** The first row shows sophisticated single-organ-specific chip designs. The second row depicts pumpless plate systems with a broad range of applications through a generic layout. The bottom row illustrates multi-organ-chips with individualized cultivation compartments. **(A)** The lung-on-a-chip hosts a barrier model containing lung epithelial and endothelial cells (reprinted with permission from Huh *et al*. [24]). **(B)** The LiverChip^®^ incorporates primary human liver cells showing extended vitality and *in vivo*-like morphogenesis over prolonged culture periods (reprinted with permission from Domansky *et al*. [89]). **(C)** The OrganoPlates^®^ allow the controlled deposition and subsequent perfusion of hydrogels (reprinted with permission from Wevers *et al*. [46]). **(D)** The microfluidic platform accommodates up to eight cell aggregates in compartments that allow communication through the medium (courtesy of Oliver Frey). **(E)** The body-on-a-chip device designed based on a PBPK model adapts compartment size and microfluidics to the human template considering technological constraints (reprinted with permission from Miller and Shuler [56]). **(F)** The Four-Organ-Chip was manufactured to mimic adsorption, distribution, metabolism and excretion – information mandatory for the toxic evaluation of any given substance. PBPK: Physiologically based pharmacokinetic.

The importance of recapitulating not only the *in vivo*-like spatial arrangement, but also interactions of different cell types was further demonstrated by Linda Griffith and her team at the Massachusetts Institute of Technology (MA, USA) (Figure 2A) [32,37,89]. They generated a hepatic capillary bed, using hepatocytes and nonparenchymal cells (including endothelial cells). By respecting the oxygen supply and demand of the cells as well as the shear stress they emulated tissue-like features. Continuous fluid flow was generated by an on-chip pump. An array of 200-μm deep and 300 × 300-μm wide through-holes was seeded with cells. Their perfusion led to the morphogenesis of hepatic sinusoidal structures. Cells with features of fenestrated endothelium could be detected even after a cultivation period of 2 weeks. To the best of our knowledge, it was the first time that delicate structures such as fenestrated endothelium could be retained under *in vitro* conditions for such a period. The liver chip models many aspects of the liver at a very high level and can support a full viral lifecycle hepatitis B. It is commercialized by CN Bio Innovations Ltd. (Hertfordshire, UK).

Epithelial barriers are another field of broad interest for toxicologists [38]. It is most important for the safety evaluations in the cosmetics industries. Generally, epithelial models provide evidence for the harmfulness of the applied substances. Furthermore, transport across barriers is regarded, which could lead to unintended local or systemic bioavailability. Absorption studies relate external exposure to internal threshold values in the context of regulatory risk assessment. The pharmaceutical industry, on the other hand, is interested in substance efficacy. Therefore, bioavailability becomes a major concern as well as first-pass metabolism in the skin. Various *in vitro* epithelial barrier models have already found applications in both research and industry as attractive alternatives to animal testing [38]. However, major challenges regarding the generation of physiologically relevant structural, mechanical, absorptive and transport properties have not yet been solved fully. The application of continuous physical stimuli on such cultures was proposed as beneficial in terms of model maturation.

Donald Ingber and his group at the Wyss Institute for Biologically Inspired Engineering at Harvard University (Boston, USA) presented a microphysiological single-organ system that reconstituted a functional alveolar–capillary interface in 2010 (Figure 2B) [24]. The device exerted cyclic mechanical strain on human alveolar epithelial cells and pulmonary microvascular endothelial cells grown on both sides of a thin, porous and highly flexible polydimethylsiloxane (PDMS) membrane. The strain resulted from the stretching of the membrane and the shear stress from flowing media. The perfusion was driven by an external pump. The device could replicate organ-level responses to physiological inflammatory stimuli, such as bacteria or cytokines. Hence, endothelial cells were activated, which led to the induction of adhesion and transmigration of primary human neutrophils flowing in the capillary channel. It effectively allows the creation of disease models and the investigation of inflammatory dynamics from a completely new perspective [39]. The microfluidic device and its derivatives have since also been applied to mimic other organs and parts of organs, such as the kidney's proximal tubule [40], the vascular endothelium [41,42], the bone-marrow [43] and the gut [44]. For the latter, cycling strain mimicking physiological peristaltic motions was exerted on a layer of Caco-2 cells. The group could show that a columnar epithelium developed and polarized rapidly. The barrier, thus, developed a high integrity toward small molecules. Mimicking the intestine was improved compared with models grown under static conditions. The device is commercially available from Emulate Inc. (MA, USA).

### Enabling multitissue interactions

Combining high cell density culture, continuous perfusion and multitissue coculture was presented by Jos Joore and Paul Vulto, managing directors of the company Mimetas (Leiden, The Netherlands) (Figure 2C) [45]. The company recently developed microfluidic 3D culture systems based on 384-well plates. The proprietary PhaseGuide™ technology allows a membrane-free, yet controlled, deposition of ECM gels. They can be arranged in orderly layers along a microchannel for medium perfusion. Loading the gels with cells results in 3D tissue models with physiologically relevant cell densities. As the gels are in direct contact with each other or the media channel, interactions without physical barriers are possible. A three-lane plate further allows the cocultivation with more cell types. The passive fluid flow using hydrostatic pressure and the industry-standard layout render the plates fully compatible with robotic pipetting and high-throughput screening. Three-dimensional networks of spontaneously active neurons and supporting glial cells were cultivated recently for up to 6 weeks in the device [46]. The physiologically more relevant culture conditions and the possibilities for high-throughput screening make the system ideal for early drug development and toxicity testing. However, the organ models incorporated are always based within ECM gels. This is of special importance regarding the fact that many pathologies are associated with changes in ECM production that have a strong impact on cell function. Hence, supplying cells with artificial, nonphysiological ECM compositions and structure might lead to cellular aberrations. The standardized plate format impedes organ-specific design constraints, such as the generation of air–liquid interface cultures or cocultures of more than two tissues.

The importance of interconnected multitissue devices and continuous organ–organ crosstalk was shown by Olivier Frey's group from the ETH Zurich and the company InSphero AG (Schlieren, Switzerland) (Figure 2D). Based on their expertise of microtissue formation and cultivation by gravity-enforced cellular self-assembly, a PDMS-based microfluidic system was developed connecting up to eight microtissue spheroids [47,48]. The 96-well plate-based system comprises several separate straight perfusion channels with open medium reservoirs at both ends. Both tissue loading and perfusion are gravity-driven by tilting the device. The use of spheroidal microtissues is advantageous in many ways. They are scaffold-free cell aggregates allowing a tissue-like arrangement of cells during aggregation and, hence, generally show an organotypic phenotype [49,50]. Using mixtures of different cell types enables heterotypic cell–cell contacts, further enhancing the tissue-like functionality and differentiated state of cells. Additionally, spheroids can be reliably produced in well-controlled sizes at moderate throughput and the spherical shape makes them easy to handle. Therefore, systems using spheroid models are robust and easy to apply. The proof of concept was performed cultivating rat liver and colorectal tumor microtissues for 8 days in the presence of cyclophosphamide. It demonstrated the importance of interconnected multitissue devices: the prodrug cyclophosphamide reduces tumor growth significantly, but only after bioactivation by the liver. This effect could only be observed in the microfluidic device. The discontinuous transfer of supernatant via pipetting from static liver microtissues treated with cyclophosphamide to tumor tissues did not affect the tumor growth significantly [48]. This again highlights the importance of continuous medium circulation and tissue interaction.

Another development from the ETH combines aggregate formation and chip technology in a single device [51]. It utilizes the hanging drop technology not only for organoid formation, but interconnected droplets also act as a network of interacting cultivation compartments. The platform allows robust and reconfigurable perfusion and effortless multi-organ dosage experiments.

Even though, the combination of various tissue models in a shared media circuit could be achieved by many research groups, *in vivo*-like tissue–tissue communications still remain an issue for most of them. The artificial media composition and lack of, for example, the nervous or lymphatic systems bear both challenges and chances. From a technical point of view, especially modeling polarized parenchyma such as the liver or organ feedback-loops like the enterohepatic circulation are unsolved endeavors.

### Targeting ADME profiles in MPS

However, not only multitissue interactions, but also physiologically relevant organ volumes and flow rates were shown to be of high value. Shuler *et al*. presented systems showing *in vivo*-like tissue–tissue mass ratios, flow rates and fluid retention times in the respective organ compartments. The devices developed in his group are based on physiologically based pharmacokinetic (PBPK)/pharmacodynamic models. Adhering to PBPK model parameters in device production is of particular importance for studying the ADME profiles of substances for the drug development process. The combination of different tissue cultures in a microfluidic system based on a PBPK model enables the emulation of time-dependent concentrations of a parental compound and its metabolites. Viravaidya and Shuler from the Cornell University in Ithaca (NY, USA) developed a four-compartment chip for cocultures of liver, lung and fat tissue models in 2004 [52]. Since then, many different systems and applications to toxicity testing have been published. Physiologically relevant data for substances such as naphthalene, doxorubicin, tegafur, atorvastatin and valproic acid, just to name a few, were obtained [53–55]. This success inspired many MPS engineers. The number of MPS published based on PBPK models is currently increasing. The most recent system of Miller and Shuler, furthermore, allows the integration of barrier organs, which had not been possible in previous versions [56] (Figure 2E). The spin-off, Hesperos (FL, USA), currently produces only a limited number of standard two-organ systems, but they offer to develop custom designs for almost any number of cell types.

Summarizing, the combination of relevant and highly sophisticated single-organ systems in a common perfusion system designed on physiologically based parameters represents a major gain in the pursuit of relevant test systems. Additionally, endothelial cells are known to establish an instructive vascular niche and induce organogenesis under both *in vivo* and *in vitro* conditions [57]. A continuous endothelial network that penetrates even through the tissue-engineered organoids will enhance the supply with oxygen and nutrients, as well as the depletion of metabolic products. Endothelial cells, furthermore, act as a barrier controlling the diffusion of small and large hydrophilic molecules and protecting subjacent parenchymal cells from nonphysiological mechanical strain. Therefore, integrating endothelial lining to the microfluidic circuit represents a further step toward *in vivo*-like cellular crosstalk and morphogenesis. Uwe Marx and his team from the Technische Universität Berlin and TissUse GmbH (Berlin, Germany) presented a microphysiological two-organ system where the closed microchannel circuit was entirely covered on all fluid contact surfaces with human dermal microvascular endothelial cells [58]. Here, a peristaltic on-chip micropump generated pulsatile shear stress in a widely adjustable range enabling long-term *in vivo*-like behavior of the endothelial cells. Furthermore, the near physiological fluid-to-tissue ratio and the closed microfluidic layout allowed for cell-mediated signaling and enriched medium to act on target cells. Strategies were also developed to establish a vascular network inside the integrated organ compartments [58,59]. Having a closed microfluidic network not only throughout the medium channels, but also vascularizing respective organ models of the device would bring many advantages. Size restrictions due to diffusion limits no longer apply to vascularized tissues. Furthermore, such vasculature is required as a barrier for the envisioned integration of an immune system.

The group further worked on the combination of four organ equivalents in a single MPS realizing the ADME profile (Figure 2F) [23]. An integrated skin biopsy resembled the dermal absorption route, while a human primary intestinal barrier model purchased from the company Matek represented an oral administration route. The introduction of barrier tissues relevant for the respective application routes are needed to model the bioavailability of a substance thoroughly. In-house assembled liver aggregates served as prime metabolic organ equivalents, and a proximal tubule barrier provided an excretory route, separating the general circuit from a primary urine compartment. Four-week viability and homeostasis of glucose concentration, lactate dehydrogenase activity and transcriptional markers could be shown. The platform paves the way to future body- or human-on-a-chip devices that have the potential to fully replace animal models in substance testing.

The endeavor to build a test system relevant for drug testing has advanced substantially during the last few years. No MPS strategy has ultimately gained broad acceptance yet, as the application possibilities are similarly broad. Whether any devices will become superior is questionable, after all. Many challenges and complex questions need to be answered, still. Some of which are addressed in the following sections.

### Finding the appropriate cultivation conditions in MPS

The development of MPS implicates new challenges, especially on the level of high-throughput applicability. What works very well on a laboratory level is not necessarily as efficient on a production scale.

Primary cell material (i.e., from isolations of native tissue), for example, often lacks donor availability and suffers from donor variability. Hence, there is the continuous pursuit of new cell sources that mimic the *in vivo* phenotype robustly. Apart from primary cells, cell lines, artificially immortalized cells or human pluripotent stem cells are often used. The latter encompass both human embryonic stem cells and induced pluripotent stem cells (iPSCs).

Cell lines are found repeatedly to be not optimal, not necessarily in terms of physiological comparability, but rather in terms of applicability. One third of all cell cultures are thought to have inter- and intra-species contaminations leading to gross misidentification and misinterpretation [60]. Simultaneously, genetic instability [60,61] and mycoplasma contamination [62] interfere with the reliability of cell lines. Their biggest asset is, however, their proliferative capabilities. For the MPS on an industrial level, cell sources should be expandable (i.e. non-phenotypical) to inoculate a number of devices. On the other hand, cells should not overgrow the MPS thereafter. All cells for multi-organ MPS should also be compatible. In other words, tissue engineers are looking for the Swiss Army knife among cell sources.

There is no shortage of innovation, though. The German companies upcyte and InSCREENeX are both reprogramming primary cells, such as hepatocytes, to enable their *in vitro* expansion [63–65]. The technology can be reversely switched on and off to prohibit expansion in the intended assay [66]. Another approach is the iPSC technology. Dedifferentiated from primary cells, such as fibroblasts, peripheral blood mononuclear cells or urinary epithelial cells, these cells are envisioned to redifferentiate into any cell type. The iPSCs are expanded for this, and differentiation is artificially induced and potentially stabilized in appropriate MPS cocultures. Furthermore, they offer the possibility of modeling diseased cells and ultimately patients (patient-on-a-chip) [67]. When opting for multi-organ devices comprising parts of the immune system, the compatibility between cells becomes a concern. Yet, an MPS with autologous cells is feasible with iPSCs from a single donor [34].

Furthermore, it should be noted that the choice of media is complicated when drafting multicellular or even multitissue systems. Every cell type is cultivated traditionally in a specialized and highly optimized medium designed mostly for fast cell growth rather than parameters relating to true physiological behavior. Growth factors, the addition of fetal serum or serum substitutes and even basal media compositions vary strongly. The application of undefined supplements, such as animal serum, is problematic in terms of lot-to-lot variability, standardization, interference with the drugs tested and the maintenance of some cells’ differentiation state (this is true especially for progenitor and stem cells). These substances are, thus, highly controversial not only among MPS engineers. Studies are carried out comparing single-tissue cultures and multitissue experiments performed under the same medium conditions. They are of great importance in understanding the models and the right choice of culture conditions. For cocultures, mixtures of cell-specific specialized media, new purpose-made media formulations or media without additives have been used so far [23,53,68]. Eventually, scientists are looking for the ubiquitous medium that fits all cell types. *In vivo* the cells micro-environments are exposed to interstitial fluids with tissue-specific compositions. Emulating this is, however, only possible with a functional vasculature. Only then, an ubiquitous medium would be the equivalent to ‘blood’ or ‘plasma’.

Similarly, the study of the materials used in the generation of the MPS and their influence on cells and (test) substances is of crucial importance. More complex systems are often composed of more than one material. Polydimethylsiloxane has been used frequently, especially among scientists, due to its low cost, ease of fabrication and biocompatibility. It finds its way increasingly into industrial applications. Nevertheless, PDMS is controversially discussed as it is known to adsorb and absorb small hydrophobic molecules and their metabolites. This impedes predicting the fraction of free compound reaching the cells [69]. Thermoplastic polymers, such as PC, PMMA and COC, do not face these issues and are used routinely in cell culture, and protocols for surface treatment are well-established. Here, other constraints such as material stiffness, gas permeability and the effort for design changes arise.

Finally, it is of prime importance to characterize the flow regime in the devices thoroughly. This is especially true when working with endothelial cells in the channels or with shear sensitive cells such as hepatocytes. The characterization of the actual flow in a microfluidic device is often disregarded, although computational models and calculations of flow behaviors often do not reflect reality. This is due mostly to oversimplifications in generating the model or to not taking all the influencing parameters into consideration. A thorough study of systems using techniques such as particle image velocimetry was shown to lead to well-characterized devices [23,58].

## Achievements in industrial adoption

The research into MPS is a very young field and still at a relatively early stage, but new exciting innovations and technologies are developing at an ever-increasing speed. Although there are many challenges and hurdles to be solved using MPS, nearly all major pharmaceutical and consumer products companies are involved in systematic feasibility studies. Some companies have even started to build up small investigational units for organ-on-chip devices in house. Others are in strong collaborations with the companies mentioned above providing commercially available systems. Emulate Inc., for example, announced a strategic research collaboration with Johnson & Johnson Innovation (NJ, USA) [70]. Using Emulates organ-on-chip platform, the human response of drug candidates is to be predicted and the drug development process improved. Similarly, CN Bio Innovations announced a research collaboration with an undisclosed pharmaceutical company to perform studies on their full viral lifecycle Hepatitis B model [71]. Cosmetics companies such as Beiersdorf AG (Hamburg, Germany) are already working with MPS models from TissUse GmbH [72]. Companies adopting these systems and using them for in-house decision-making during the drug development process are already currently producing high-value data relevant for later qualification studies. The widespread use of the various MPS platforms will lead to a greater familiarity with and understanding of the benefits and limitations of the technology. Showing that the high-quality data derived from these systems are consistent with those derived from other approaches (especially human data) and, furthermore, using them to answer questions that cannot be addressed in animal models will provide further testimony for their reliability.

European flagship projects such as the EU-ToxRisk further support the development of new approaches for mechanism-based toxicity testing and risk assessment. The final goal is a paradigm shift in toxicology toward an animal-free chemical safety assessment. An international consortium of 39 partners has been funded by the European Commission since 2016 to integrate new concepts such as MPS-based systems for regulatory chemical safety assessment. This evaluation of MPS under European programs and the submission of reliable data will aid not only industrial adoption, but also regulatory acceptance. An expert report recently depicted that the industrial adoption period started in 2015 will finally lead the way for regulatory acceptance in 2020 [34].

## Regulatory acceptance through validation

It has always been a challenge for regulators across the world to meet the legislative requirements. Further demands in intensified safety assessments will complicate substance authorization. *In vitro* methods are intended to handle future risk evaluation. Alternative methods need to fulfill the ‘fitness-for-purpose’ criteria for regulatory acceptance [12]. Consequently, the EMA gave out three criteria [73]: first, a method needs to be valid, meaning it has a standardized protocol and clear readouts. Further, it needs to be reliable, relevant and validated by recognized institutions. Second, the new method should generate new and meaningful data or replace an existing method with the same or better results. Third, the new method must have been applied in parallel, but independently to an accepted method in a regulatory decision-making process.

To achieve these criteria, first, the qualification of the equipment operating the device according to standard installation, operation and performance qualification is required. Second, the generation of reliable, reproducible and robust data needs to be assured by the adherence to the rules of Good Cell Culture Practice [74], and the use of qualified cell and tissue sources. The improvement of existing models and assays toward a closer recapitulation of *in vivo* physiology was also recommended recently [75]. Defining the critical regulatory gaps which can be addressed by the MPS is essential to define the biological and functional features of the model system. In addition, the library of reference compounds for qualification should be linked directly to the regulatory question being addressed. Similarly, the ‘scientific basis’ needs to be properly described. This includes the biological and physiological processes modeled by the test system and thought to be relevant for the substances’ adverse effect.

The final validation roadmap might then comprise two different ways depending on the segment and aim of the model. Systems aiming for safety and efficacy testing during drug development might be qualified through the US FDA drug development tool (DDT) Qualification program. This is especially true for systems requiring fast track validation, such as those MPS models targeting compounds or diseases that cannot be tested using techniques currently available. This applies especially to novel biopharmaceuticals specially designed for humans and irrelevant in test animals. Here, a clear description of the manner and purpose of use of the systems as well as its limitations is mandatory. Currently, the FDA is implementing DDT qualification programs for biomarkers and animal models. However, this concept can also be applied to other tools proposed for use in regulatory decision-making, including MPS. Furthermore, the validation of an individual biomarker under the Biomarker Qualification Program on a specific MPS may be a key step toward qualification and acceptance as a tool for therapeutics development. Finally, qualifying an MPS for a specific context of use as a DDT will eventually lead to a tool which may be considered product-independent and may be used for efficacy testing in development programs for multiple investigational drugs for the same disease or condition targeted.

The MPS that aim to replace animal or *in vitro* models in cosmetics and chemicals safety assessment need to oblige the respective OECD guidelines. The process of identifying the hazard potential is highly standardized. It is recommended that MPS adhere to the OECD guidance document on the validation and international acceptance of new or updated test methods for hazard assessment [76]. Here, high-quality data from MPS that are consistent with results derived from other approaches will provide initial evidence for their reliability. However, care must be taken when comparing results gained from human-based MPS with animal studies as the latter might lack sensitivity or specificity. If we, today, validate models based on existing models that are not 100% reliable, we might pass old flaws to new methods. Cross-validation trials and the assessment of biological baselines of MPS-based approaches would be constructive. Obviously, the comparison to the human situation would be the most precise. However, “human data relating to chemical effects (…) are normally not readily available or need at least to be derived from highly uncertain information (e.g., epidemiological data), involving moreover expert judgement” [12]. The demonstration that the new devices can be used to identify compounds that have failed in clinical trials will generate more confidence in modern MPS technologies.

## Living up to high expectations

The expectations of MPS in risk assessment are high, and the demand is even higher. With the European Cosmetics Directive [20], the European REACH regulation [21] and the new amendment to the US Toxic Substances Control Act [77], animal alternatives are being promoted even by legislators. Simultaneously, vast testing especially of existing chemicals is demanded [8]. However, expectations and reality come to a hard clash when toxicologists require from new technologies what current standards cannot fulfill. A study analyzing noncarcinogenic endpoints in mouse and rat experiments came to the conclusion that the results gained from one species cannot predict with high certainty the outcome in the other species and *vice versa* [78]. Depending on the organ regarded, the results could be even completely contradictory. The Center of Alternatives to Animal Testing and partners looked into *in vivo* skin sensitization and eye irritation data available through the REACH database [79,80]. They found that the intrareproducibility of the tests never exceeded 95%. The reproducibility even lay at only 73% for positive-evaluated eye irritants. The inter-reproducibility can be as low as 77% and as high as 92%. Discrepancies between the tests are also admitted in the respective OECD guidelines [81]. As pointed out by Browne *et al*. [82]: “Recognizing this variability [of *in vivo* experiments] is important because it sets realistic expectations as to the performance of any alternative method. (…) It is unrealistic that an alternative method should predict both the true positives as well as account for the associated *in vivo* experimental variability.”

## Toward a human-on-a-chip

The MPS that have been described above and that are already on the market have great potential in the early elimination of toxic drug candidates before moving to animal testing. However, these models only answer specialized questions concerning drug distribution, metabolism and effects on predefined organ systems. A systemic model at organismal-level is required aiming for a fully new substance testing paradigm which eliminates the need for animal testing.

**Figure 3.**
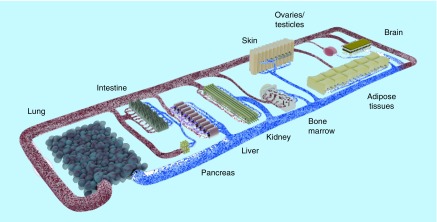
**Concept of a human-on-a-chip.** The pursuit of the most important bodily functions will lead to a miniature organism on a chip. Common tests conducted in rodents should be realizable with this device. Therefore, the products of the organoids (e.g., urine) need to be discharged in separate compartments. Oral, dermal and intravenous uptake routes, and through inhalation, need to be possible. The transparent device enables optical analysis. Incorporated electrodes will assess barrier resistance, electrophysiological data and key parameters in the supernatants.

The goal here is not only to cocultivate several physiologically relevant organ models in a combined media circuit, but also to obtain a self-contained organismal homeostasis. To achieve this, it was postulated previously that at least the following ten systems should be present and show *in vivo*-like interaction: circulatory, endocrine, gastrointestinal, immune, integumentary, musculoskeletal, nervous, reproductive, respiratory and urinary (Figure 3) [34].

Of course, several obstacles, especially at the biological–technical interface, need to be resolved. Exemplarily, so far none of the numerous liver-on-a-chip devices has shown an effective segregation of bile into a secondary compartment outside the blood circuit. Similarly, the fine structure of spleen or kidney could not be artificially recreated. Three-dimensional bioprinting might provide a solution to many of these problems. It allows the reproductive and ordered deposition of diverse cell types and materials, enabling the patterning of complex organoid fine structures and gradients. Nevertheless, spatial resolution, matrix interactions and cell densities need to improve [83].

### Enhancing reliability by improving readouts

The integration of various sensors and actuators enhances the degree of control over these cultures and enable physiologically relevant electro–mechano–biochemical signaling. The readout of the electrical activity of neuronal and cardiac cells, for example, and the electrical stimulation of muscle tissue can be performed with the help of microelectrodes integrated into MPS [84,85]. Similarly, transepithelial electrical resistance (TEER) measurements were used to study the integrity of barrier models in MPS [86]. Sensors based on fluorescent indicators as well as label-free microsensors for pH, dissolved gases and temperature, furthermore, allow for online monitoring of the culture conditions. The integration of mechanical coupling is particularly important for the maintenance of differentiated cells derived from organs that perceive constant physical stimuli. That includes not only shear stress on endothelial cells generated by flowing media, but also directed expansion and compression forces relevant for lung, bone and cartilage [24,58]. Similarly, skin and the GI tract are prone to relevant mechanical loads. Sophisticated MPS have been developed to realize these *in vitro* [87,88]. However, combining these approaches into one microfluidic device remains challenging.

The complexity of MPS and human-on-a-chip devices requires the retrieval of as much information as possible from each single experiment. Therefore, high-content end point analyses, such as RNA sequencing, supernatant metabolite analysis and biomarker measurements, can be performed to characterize the differentiated status of cells and tissues and provide quality control parameters. Combining such readouts with noninvasive sensor-based low content online measurements and microscopic readings can result in a deep insight into tissue biology during multi-organ or human-on-a-chip culture. The complexity is also a reason why interlab reproducibility and transferability has to be tested thoroughly. A high degree of automation is envisioned in the near future to make the systems robust and assays scalable toward industrial needs.

## Conclusion & future perspective

The MPS have evolved recently combining 3D *in vitro* cell cultures in microfluidic devices with modern mechanical or electrical actuators and sensors. These systems have already shown major improvements which are physiologically relevant. Furthermore, the single- and multi-organ devices presented allow for a better preselection of effective drugs and more accurate prediction of drug-induced organ toxicity. As long-term organ cultures were shown to be possible, repeated dose toxicity assays are feasible. To date, models for the most prominent delivery routes – oral, dermal and inhalation – are under evaluation in MPS systems and devices allowing for ADME profiles. The experience acquired from these MPS and future developments will eventually lead toward the generation of a miniature organism on a chip.

The intensified adoption of MPS in the pharmaceutical industry should lead within the next 5 years to a faster and more precise selection of targets and compounds during drug discovery and lead optimization. In a next step, the industrial adoption and data generation using MPS should trigger a consecutive regulatory acceptance.

Once human-on-a-chip devices allow for predictive systemic testing, it will no longer be required to involve animals in preclinical development and healthy volunteers in Phase I testing. Although it is an ambitious project, the development of human-on-a-chip systems has been recognized as a promising route toward a paradigm shift in the drug development process.

**Executive summary**

- The fast progress in microphysiological systems (MPS) development has raised expectations that it might revolutionize substance development and risk assessment. These systems are capable of mimicking the physiological interaction of various interconnected organ models emulating organismal functionality and response to substances.
- Microphysiological systems in general are designed to support a physiological environment for *in vitro* cultures and emulate human biology at the smallest acceptable scale. Their ability to host 3D organoid constructs in a controlled microenvironment with mechanical and electrophysiological stimuli has been shown previously.
- A 3D coculture of various cell types, as well as physical stimulation of organ models in MPS were shown to enhance physiological relevance.
- Microphysiological systems show broad application possibilities. Hence, the systems reported have a similarly broad technological and biological spectrum. For example, the mode of pumping, the format of the device or the complexity of organ models integrated vary.
- Founding MPS on physiologically based pharmacokinetic/pharmacodynamic models allows one to describe the absorption, distribution, metabolism and excretion profile of a compound quantitatively.
- The integration of a fully closed microvascular network, covering all channel surfaces and penetrating organ models would represent a major step forward toward a self-sustained systemic model system and the utilization of a blood surrogate.
- Primary issues that need to be addressed are the choice of cell source and finding the optimal media composition for multi-organ MPS.
- Major pharma and cosmetics companies are already testing MPS in house. Furthermore, European flagship programs target MPS qualification.
- So far, no MPS achieved regulatory validation. Still, various strategies to facilitate regulatory acceptance exist.
- To achieve the final target of a systemic human-on-a-chip model at organismal-level major questions need to be answered. Still, it holds great potential for a fully new substance testing paradigm eliminating or reducing the need for animal trials.
